# Editorial: Response to cardiac resynchronization therapy

**DOI:** 10.3389/fcvm.2023.1297343

**Published:** 2024-01-05

**Authors:** Annamaria Kosztin, Alexander Maass, Igor Diemberger

**Affiliations:** ^1^ Department of Cardiology, Heart and Vascular Center, Semmelweis Univeristy, Budapest, Hungary; ^2^ Department of Cardiology, University Medical Center Groningen, University of Groningen, Groningen, Netherland; ^3^ Institute of Cardiology, Department of Medical and Surgical Sciences, University of Bologna, Policlinico S.Orsola-Malpighi, Bologna, Italy; ^4^ UOC di Cardiologia, IRCCS Azienda Ospedaliero-Universitaria di Bologna, Dipartimento Cardio-toraco-vascolare, Bologna, Italy

**Keywords:** cardiac resynchronizaion therapy, cardiac resynchronization biventricular defibrillator, conduction system pacing, remote monitoring, heart failure - implantable cardioverter-defibrillator - multipoint pacing - battery longevity

## Abstract

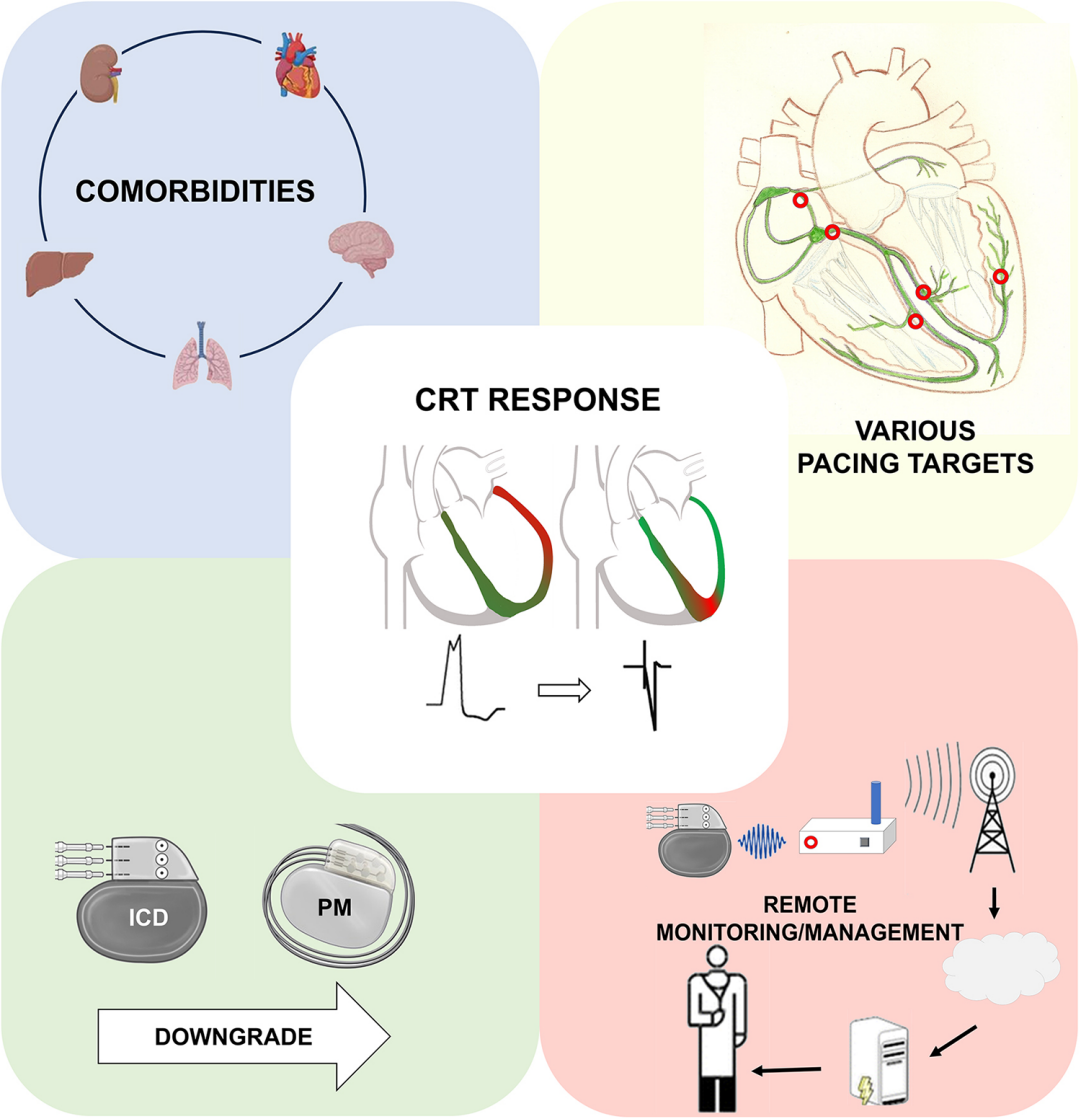

**Editorial on the Research Topic** Response to cardiac resynchronization therapy

## Introduction

Since the implantation of the first pacemakers in the 1960s, rapid advancements in biomedical technologies have led to the development of Cardiac Resynchronization Therapy (CRT) in the 1990s. Landmark randomized controlled trials conducted in the early 2000s demonstrated the reduction of mortality and morbidity by the use of *de novo* CRT in heart failure (HF) patients with a reduced ejection fraction (HFrEF) and intrinsic, wide QRS complex (1, 2).

Moreover, now evidence on CRT upgrade with hard outcome is also available for patients with HFrEF and wide, paced QRS (3). Patients with a device upgrade from a conventional pacemaker or an implantable cardioverter defidrillator (ICD) with an intermittent or permanent right ventricular pacing also show a clear benefit (heart failure hospitalization, all-cause mortality and echocardiographic response) from adding an extra left ventricular (LV) lead, providing a relevant population for CRT implantation (3).

However, CRT still presents challenges, with a relatively high rate of non-responders and potential procedural complexities due to anatomical variations among patients. After more than two decades of CRT use, to increase the rate of responders,

(a) Patient selection has become more precise and optimal e.g. with using risk stratification (4, 5) and response scores (6),
(b) Implantation techniques have emerged in the field of pacing, such as Conduction System Pacing (CSP), and
(c) During the follow-up, strict monitoring of patients through remote systems are all leading to a better outcome of our patients and a decreased risk of adverse clinical events.

However, there remain unmet needs and gaps in the evidence related to the optimal device types at the time of implantation and subsequently at generator replacement (CRT with or without defibrillator), the use of new techniques, prediction of our patients’ outcome, strict follow-up and early selection of those with a poor response.

### Conduction system pacing

Data on CSP is limited compared to CRT so far, but it may be on course to become a viable option for the treatment of HF alongside CRT, as suggested by an extensive review conducted by Moustafa et al. CSP comprises various pacing sites, such as His-bundle pacing (HBP) and left bundle branch pacing (LBBAP), which are safe and feasible alternatives to CRT. However, several disadvantages may arise, including higher pacing thresholds or dislocations, reduced battery life, and challenges related to extended procedure and fluoroscopy time, or unknow issues at lead extractions (7).

Additionally, assessments of long-term outcomes and hard endpoints comparing CSP to CRT through randomized control trials (RCTs) are still awaited.

However, with the advent of new pacing options and technologies to reduce electromechanical dyssynchrony, biventricular pacing can no longer be solely attributed to CRT. Marcantoni et al. propose the necessity of new terminologies, as biventricular pacing can now be achieved through CSP, CRT, and their combinations (e.g., LOT-CRT, HOT-CRT).

### Remote monitoring

Remote monitoring (RM) enhances the efficacy of healthcare, facilitates informed decision-making, and holds the potential to detect early signs of heart failure (HF) decompensation, ultimately reducing the burden of major cardiovascular events and the need for in-person visits. As a result, RM not only eases the workload of caregivers but also plays a pivotal role in preventing HF-related hospitalizations and death (8).

In a study conducted by Marini et al., it was reported that RM significantly decreased the number of hospitalizations for cardiovascular (CV)-related reasons and all-cause mortality when compared to standard monitoring (SM) over a 2-year follow-up period. Interestingly, there was no difference in the number of in-office visits between the two treatment groups, as the RM group had received device alerts. Furthermore, remote monitoring led to a notable reduction in the overall cost of care, encompassing both inpatient and outpatient care.

Despite the initial implementation costs associated with RM, along with the increased number of in-office visits triggered by RM alerts, the total cost of care decreased due to the reduced rate of hospitalizations for CV-related diseases.

### Risk stratification and the necessity of an implantable cardioverter defibrillator in cardiac resynchronization therapy candidates

The implantation of a primary prevention ICD with CRT in patients with multiple comorbidities, particularly in cases of severe renal insufficiency, is often discouraged. These patients are more likely to experience premature mortality due to causes unrelated to sudden cardiac death (SCD) and may also have a higher risk for inappropriate shocks due to hyperkalaemia. Goldenberg et al. assembled a patient cohort from the MADIT-CRT and RAID trials to investigate the relevance of primary prevention ICDs in patients with chronic kidney disease (CKD). Their study revealed that patients with more severe CKD (Stage 3b to Stage 5) were less prone to ventricular arrhythmias (12.3% vs. 23.5%) but had a five-fold higher rate of non-arrhythmic mortality compared to those with less advanced CKD (Stage 1 to 3a). Future randomized trials are imperative to determine whether CRT-P is non-inferior to CRT-D in patients with advanced CKD.

### Downgrading CRT-D devices to CRT-P

The controversy surrounding the use of ICDs is not limited to the initial implantation of CRT but should also be reevaluated during generator exchange. In cases where patients exhibit significant reverse remodeling and clinical improvement, the necessity of defibrillator therapy may diminish, especially in an era marked by the availability of potent pharmacological treatments for HF (e.g., SGLT-2 inhibitors or ARNI). Frey et al. conducted a study involving super-responder patients who underwent either downgrading to CRT-P at the time of generator exchange or continued CRT-D therapy. During their follow-up, none of the downgraded patients experienced fatal ventricular arrhythmic (VA) events, while two patients in the CRT-D group suffered VAs. It is worth noting that 74% of all patients had non-ischemic etiology, which could potentially introduce bias into their results. Further analyses are required, and, for now, it is advisable to adopt an individualized decision-making approach with each patient. As of yet, no adapter is available for DF-4 ICD electrodes and downgrade is restricted to the old-fashioned ICD leads with a DF-1 connector in addition to an IS-1 pace-sense part. At the time of implantation, the future necessity of device downgrade needs to be taken into account and if used in the correct fashion this can translate to an increasing number of device downgrades (9).
